# Antibiotic Stimulation of a *Bacillus subtilis* Migratory Response

**DOI:** 10.1128/mSphere.00586-17

**Published:** 2018-02-21

**Authors:** Yongjin Liu, Steven Kyle, Paul D. Straight

**Affiliations:** a Biochemistry and Biophysics Department, Texas A&M University, College Station, Texas, USA; b Interdisciplinary Program in Genetics, Texas A&M University, College Station, Texas, USA; University of Iowa

**Keywords:** *Bacillus subtilis*, *Streptomyces venezuelae*, antibiotics, chloramphenicol, competition, hormesis, ribosome, sliding motility

## Abstract

Competitive interactions between bacteria reveal physiological adaptations that benefit fitness. *Bacillus subtilis* is a Gram-positive species with several adaptive mechanisms for competition and environmental stress. Biofilm formation, sporulation, and motility are the outcomes of widespread changes in a population of *B. subtilis*. These changes emerge from complex, regulated pathways for adapting to external stresses, including competition from other species. To identify competition-specific functions, we cultured *B. subtilis* with multiple species of *Streptomyces* and observed altered patterns of growth for each organism. In particular, when plated on agar medium near *Streptomyces venezuelae*, *B. subtilis* initiates a robust and reproducible mobile response. To investigate the mechanistic basis for the interaction, we determined the type of motility used by *B. subtilis* and isolated inducing metabolites produced by *S. venezuelae*. *Bacillus subtilis* has three defined forms of motility: swimming, swarming, and sliding. *Streptomyces venezuelae* induced sliding motility specifically in our experiments. The inducing agents produced by *S. venezuelae* were identified as chloramphenicol and a brominated derivative at subinhibitory concentrations. Upon further characterization of the mobile response, our results demonstrated that subinhibitory concentrations of chloramphenicol, erythromycin, tetracycline, and spectinomycin all activate a sliding motility response by *B. subtilis*. Our data are consistent with sliding motility initiating under conditions of protein translation stress. This report underscores the importance of hormesis as an early warning system for potential bacterial competitors and antibiotic exposure.

**IMPORTANCE** Antibiotic resistance is a major challenge for the effective treatment of infectious diseases. Identifying adaptive mechanisms that bacteria use to survive low levels of antibiotic stress is important for understanding pathways to antibiotic resistance. Furthermore, little is known about the effects of individual bacterial interactions on multispecies communities. This work demonstrates that subinhibitory amounts of some antibiotics produced by streptomycetes induce active motility in *B. subtilis*, which may alter species interaction dynamics among species-diverse bacterial communities in natural environments. The use of antibiotics at subinhibitory concentrations results in many changes in bacteria, including changes in biofilm formation, small-colony variants, formation of persisters, and motility. Identifying the mechanistic bases of these adaptations is crucial for understanding how bacterial communities are impacted by antibiotics.

## Abstract (precis)

Antibiotic resistance is a major challenge for the effective treatment of infectious diseases. Identifying adaptive mechanisms that bacteria use to survive low levels of antibiotic stress is important for understanding pathways to antibiotic resistance. Furthermore, little is known about the effects of individual bacterial interactions on multispecies communities. This work demonstrates that subinhibitory amounts of some antibiotics produced by streptomycetes induce active motility in *B. subtilis*, which may alter species interaction dynamics among species-diverse bacterial communities in natural environments. The use of antibiotics at subinhibitory concentrations results in many changes in bacteria, including changes in biofilm formation, small-colony variants, formation of persisters, and motility. Identifying the mechanistic bases of these adaptations is crucial for understanding how bacterial communities are impacted by antibiotics.

## INTRODUCTION

Bacteria have various mechanisms to maintain fitness under conditions of competitive stress. Examples of competitive fitness mechanisms include type VI secretion systems or contact-dependent inhibition (1–3) and chemical mechanisms as exemplified by antibiotics and other specialized metabolites (4–8). Resistance to a specific challenge also promotes competitive fitness through chemical or genetic modifications to a target or a toxin (9–11). Additionally, adaptations to the physiology of cells within a population or community may alter susceptibility to various competitive stresses. For instance, bacteria may induce biofilm formation (8), enter a persister state (12), or activate a specialized form of metabolism in response to competitors (13–16). One adaptive mechanism available to many species is motility, which imparts to bacteria the ability to physically relocate in the event of a competitive challenge (17–21). In some cases, the response may be chemotactic, manifesting as avoidance of a toxic substance through receptor activation of motility controls. Other sensing or stress mechanisms that activate mobility are not well defined. In one example, swimming and swarming motility are enhanced when *Pseudomonas aeruginosa* is exposed to the antibiotic tobramycin, but the underlying mechanism is unknown (22). How bacteria sense and respond to antibiotic stress is of particular interest for understanding the development of antibiotic resistance. Indeed, a connection between motility and antibiotic resistance, where resistance is elevated in some motile populations of bacteria, has been found (23, 24).

*Bacillus subtilis* serves as a model for motility of Gram-positive bacteria. *B. subtilis* has three described mechanisms of motility: swimming, swarming, and sliding (25–29). Swimming and swarming motility are driven by the action of flagella, which provide propulsion to the bacteria. Swimming *B. subtilis* use multiple, peritrichous flagella to move as single cells through aqueous media. When the surrounding medium is sufficiently viscous, *B. subtilis* cells join into rafts that use swarming motility to migrate across surfaces under the power of flagella extending from multiple cells (26). The third type of movement, sliding, is flagellum-independent motility driven by growth. Sliding is currently understood to depend upon multiple factors, including potassium, production of the lipopeptide surfactin, exopolysaccharides (EPS), and extracellular proteins BslA and TasA (29–31). At the vanguard of a sliding population, combinations of surfactin-producing cells and EPS-producing cells cooperate to generate “van Gogh” bundles characteristic of sliding on specialized media (30). The coordinated activities of cell subpopulations within a colony indicates orchestration of multiple events to promote cooperative sliding. Some regulatory functions that contribute to sliding mobility have been described previously (31), but the overall process is less clearly understood than either swimming or swarming motilities. Additionally, other competitive functions may be coordinately controlled with mobilization of cells. In combination with resistance functions, a mobilized bacterial population potentially possesses multiple advantages for competitive fitness.

Here we describe a competitive interaction between *Streptomyces venezuelae* and *B. subtilis*. We observed that, under conditions of coculture with *S. venezuelae*, *B. subtilis* activates a motile response. First, we identified the type of motility as sliding. Second, we extracted an inducer of sliding motility from agar plates of *S. venezuelae* and, to our surprise, identified the inducer as the antibiotic chloramphenicol (Cm). At subinhibitory concentrations, many antibiotics possess stimulatory activity, triggering a response in exposed bacteria. This phenomenon, known as hormesis, has been studied for many species and antibiotics (32, 33). Prior studies have shown that subinhibitory concentrations of antibiotics induce responses in exposed bacteria, including changes in transcription, biofilm formation, persistence, and altered virulence (5, 22, 33–37). While tobramycin was previously seen to enhance motility of *P. aeruginosa*, induction of motility in an otherwise nonmotile population has rarely been reported (19, 20, 22, 34, 38). In addition to chloramphenicol, we found that other antibiotics that target the ribosome also induce motility. Targeted analysis of genes associated with translation stress and antibiotic resistance suggested that the sliding response occurs when ribosome function is perturbed. On the basis of these observations, we suggest that *B. subtilis* engages a programed motile response to competitive stress that results from subinhibitory antibiotic interference with protein translation.

## RESULTS

### Competitive interaction with *Streptomyces venezuelae* induced mobilization of *Bacillus subtilis* NCIB 3610.

**FIG 1:**
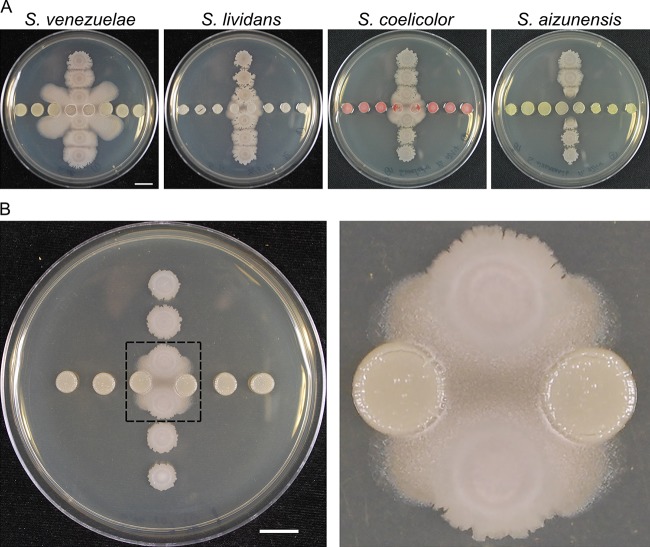
*S. venezuelae* induces *B. subtilis* mobilization. (A) Different species of *Streptomyces* were cultured with *B. subtilis* to identify patterns of interaction. *Streptomyces* species were spotted in the horizontal line, and *B. subtilis* was in the vertical line. Pictures were taken at h 40. (B) *S. venezuelae* (horizontal spots) induced proximal *B. subtilis* (vertical spots) to migrate across the agar surface, while this migration was not observed in the distal spots. The right panel presents an enlarged view, highlighting the mobile region inside the dashed box. The picture was taken at h 18. Bars, 1 cm.

To identify patterns of interaction of *B. subtilis* NCIB 3610 with *Streptomyces* species, we plated pairs of the two species on rich agar media in a cross-wise pattern. The spotting pattern enables assessment of differential interactions determined by the proximity of competing species. *Streptomyces venezuelae* reproducibly induced proximal spots of *B. subtilis* to initiate a migration across the agar surface (Fig. 1A). In contrast, other species (e.g., *Streptomyces lividans* and *Streptomyces coelicolor*) also induce mobilization but do so with delayed timing and to a lesser extent than *S. venezuelae* (38). In some cases (e.g., *Streptomyces aizunensis* and *Streptomyces* sp. strain Mg1), mobilization is not observed, either due to lack of induction or because the lysis observed upon coculture disrupts mobilization (19) (Fig. 1A). On the basis of the pattern and the robust reproducibility of *B. subtilis* mobility induced by *S. venezuelae*, this interaction was investigated further.

Two features evident in the observed pattern indicated a complex interspecies interaction. First, the initial migration of *B. subtilis* across the agar surface is oriented toward the competitor *S. venezuelae* (Fig. 1B) (see Movie S1 in the supplemental material). The surface characteristics change for the *B. subtilis* mobile population, which acquires a rough appearance in comparison to the parent spot. The difference in colony texture indicates a major transition in cellular organization, reminiscent of swarming motility or biofilms (26, 39). Second, as the mobilized population progresses outward toward adjacent *S. venezuelae* patches, it appears to be repelled (Fig. 1A) (Movie S1). The observed patterns of migration toward *S. venezuelae* suggested that *B. subtilis* responds to the presence of diffusible substances produced by *S. venezuelae*. On the basis of the observed interaction pattern, we sought, first, to define the type of motility used by *B. subtilis* and, second, to identify inducing substances produced by *S. venezuelae*.

10.1128/mSphere.00586-17.9 MOVIE S1  Competitive interaction between *B. subtilis* and *S. venezuelae*. Spots of each bacterial species on agar media were captured by time-lapse video over 72 h and reveal the pattern of sliding motility exhibited by *B. subtilis*. Initially, *B. subtilis* moved toward the proximal *S. venezuelae* spots (up to ~36 h). Continued culture showed that the sliding population of *B. subtilis* progressed outward and that the population deflected away from the *S. venezuelae* population (up to 72 h). The agar plate was 8.4 cm in diameter. Download MOVIE S1, MOV file, 5 MB. Copyright © 2018 Liu et al. 2018 Liu et al. This content is distributed under the terms of the Creative Commons Attribution 4.0 International license.

### *S. venezuelae*** induces flagellum**-**independent sliding motility in** *B. subtilis**.***

**FIG 2:**
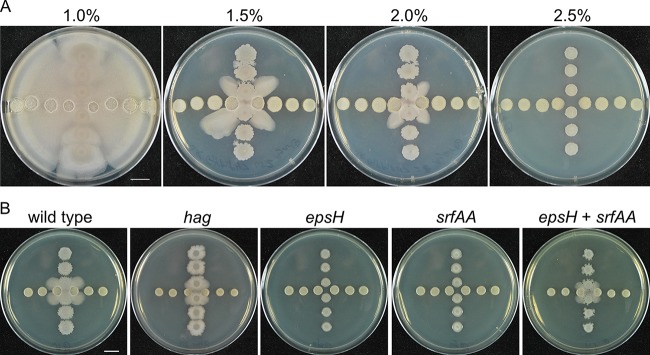
Identification of *S. venezuelae*-induced mobility as sliding. *S. venezuelae* was spotted in the horizontal line in both panels A and B. Pictures were taken at h 48. (A) The mobilization induced by *S. venezuelae* was observed at up to 2% agar. (B) Different *B. subtilis* mutants were cultured with *S. venezuelae*. The mobility of *hag* mutant was induced but was not observed in either *epsH* mutants or *srfAA* mutants. However, when *epsH* and *srfAA* were mixed, the mixture was able to mobilize upon challenge with *S. venezuelae*. Pictures were taken at h 24. Bars, 1 cm.

To understand the molecular basis for migration of *B. subtilis*, multiple approaches were used to identify the type of motility induced by *S. venezulae*. *Bacillus subtilis* migration depends upon the viscosity of the surrounding medium. For instance, increasing agar concentrations limit the type of motility available. Agar concentrations above 0.3% (wt/vol) and 1% (wt/vol) prevent swimming and swarming motilities, respectively (25). To characterize the motility of *B. subtilis* in response to *S. venezuelae*, we spotted both species onto solid media of different agar concentrations. The motile response to *S. venezuelae* persisted at agar concentrations of up to 2% (wt/vol), limiting the possibility of identifying swimming or swarming as a basis for motility (Fig. 2A). A third type of motility, sliding, has been demonstrated on specialized media with agar or agarose concentrations typically less than 1% (wt/vol) (29–31, 40). However, because the induced migration of *B. subtilis* was observed at up to 2% (wt/vol) agar, a concentration which has not been tested in sliding motility experiments, additional experiments were performed to determine the type of motility.

To identify genetic requirements for motility, we tested mutant strains that are defective for different types of motility. We first used a strain deficient in the flagellin protein (Δ*hag*) which is incapable of producing flagella (41, 42). Although the strain with the Δ*hag* mutation displayed defects in colony morphology and motility, possibly due to overproduction of surfactin, induction of migration by *S. venezuelae* was clearly observed using this strain (26) (Fig. 2B). Therefore, the motility observed includes a flagellum-independent component. Sliding motility depends on extracellular polysaccharides (EPS) and surfactin (28–30). Mutant strains that are unable to produce a poly-N-acetylglucosamine component of EPS (*epsH*) or surfactin (*srfAA*) were unable to migrate in response to *S. venezuelae* (43) (Fig. 2B). Because EPS and surfactin are both extracellular products, the single mutant strains were combined to test for extracellular complementation (30). When the *B. subtilis epsH* and *srfAA* mutant strains were mixed and competed with *S. venezuelae*, the migration was restored. Consistent with those results, we identified disruptions in *eps* and *srf* genes in a transposon mutagenesis screen for *B. subtilis* strains that failed to exhibit migration (see Table S3 and Text S1 in the supplemental material). Together, these results strongly suggest that *B. subtilis* sliding motility is induced by *S. venezuelae*.

10.1128/mSphere.00586-17.1 TEXT S1  Supplemental Methods. Download TEXT S1, DOCX file, 0.1 MB. Copyright © 2018 Liu et al. 2018 Liu et al. This content is distributed under the terms of the Creative Commons Attribution 4.0 International license.

### Identification of an inducing metabolite produced by *S. venezuelae*.

**FIG 3:**
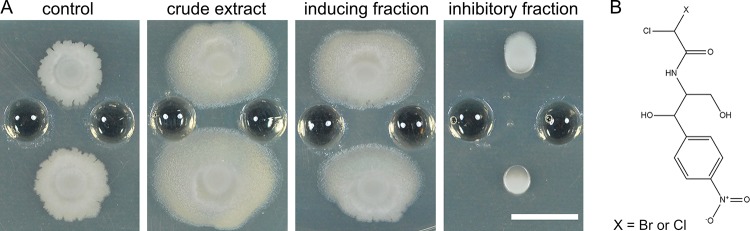
Identification of monobromamphenicol as a sliding inducer. (A) Crude extract from *S. venezuelae* agar plates was loaded into the wells near *B. subtilis* and induced robust sliding motility compared with the medium-only control. All time-based HPLC fractions were collected and tested for activity. One fraction had the sliding inducing activity, and one fraction had the growth inhibitory activity. Pictures were taken at h 24. (B) The inducing fraction was brominated chloramphenicol (X = Br [monobromamphenicol]). The inhibitory fraction was chloramphenicol (X = Cl). Bar, 1 cm.

The observed patterns of migration suggested that *S. venezuelae* produces a substance or substances that induce sliding by *B. subtilis*. One hypothesis is that a metabolite or enzyme secreted by *S. venezuelae* activates a specific response in *B. subtilis* cells, leading to the observed sliding motility. To identify an inducer substance, we extracted agar media after culturing *S. venezuelae* in isolation. Concentrated crude extracts were then added to wells adjacent to *B. subtilis* colonies to determine whether inducing activity was present (Fig. 3A). Comparison to a medium-only control revealed robust inducing activity in the crude extract, which was subsequently fractionated using solid-phase extraction first and then high-performance liquid chromatography (HPLC) (see Fig. S1 in the supplemental material). We then collected time-based HPLC fractions and screened for activity on agar plates. The inducing activity was abundant in a single fraction (Fig. 3A). The active fraction was analyzed by liquid chromatography-tandem mass spectrometry (LC-MS/MS) to identify candidate inducer metabolites (Fig. S2A and B). An abundant signal identified by MS1 and MS2 analysis was consistent with that of monobromamphenicol, a variant of chloramphenicol where one chlorine atom is replaced by a bromine atom (44) (Fig. 3B).

10.1128/mSphere.00586-17.2 FIG S1  HPLC trace of 40% methanol fraction from crude extracts. Crude extract was further fractionated. The active 40% methanol fraction was applied to HPLC for further separation at a wavelength of 254 nm. The peak corresponding to the inhibitory fraction is labeled with a star. The peak corresponding to the inducing fraction is labeled with a pound sign. Download FIG S1, TIF file, 0.2 MB. Copyright © 2018 Liu et al. 2018 Liu et al. This content is distributed under the terms of the Creative Commons Attribution 4.0 International license.

10.1128/mSphere.00586-17.3 FIG S2  Identification of monobromamphenicol and chloramphenicol by HPLC-MS/MS. (A) The mass and the isotope profile are consistent with those of monobromamphenicol. Different forms of parent ions are labeled in the MS1 spectrum. (B) The identity of monobromamphenicol was further confirmed by analysis of the fragment ions in the MS2 spectrum. (C) The mass and the isotope profile are consistent with those of chloramphenicol. Different forms of parent ions are labeled in the MS1 spectrum. (D) The identity of chloramphenicol was further confirmed by analysis of the fragment ions in the MS2 spectrum. Download FIG S2, TIF file, 0.5 MB. Copyright © 2018 Liu et al. 2018 Liu et al. This content is distributed under the terms of the Creative Commons Attribution 4.0 International license.

**FIG 4:**
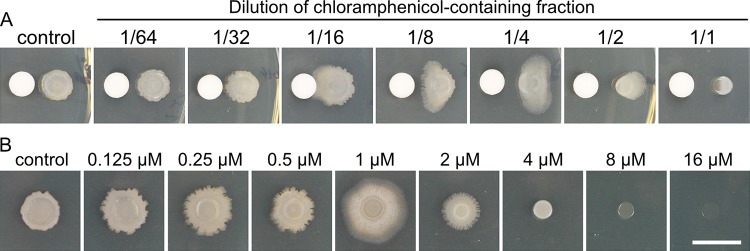
Chloramphenicol induced *B. subtilis* sliding at subinhibitory concentrations. (A) The chloramphenicol fraction was 2-fold serially diluted, and 10 μl of each dilution was applied onto a filter paper disc 0.6 cm away from *B. subtilis*. The control was the 40% (vol/vol) methanol solvent. (B) Pure chloramphenicol was serially diluted and added to the agar plate. At 1 µM, the maximal sliding response was induced. The control was the plate without chloramphenicol. Pictures were taken at h 24. Filter disc diameter, 0.6 cm. Bar, 1 cm.

*Streptomyces venezulae* is well known as a producer of chloramphenicol and is the species from which the antibiotic was originally identified (45, 46). Brominated derivatives have been produced synthetically and by feeding bromine to cells but are not described as natural products of *S. venezuelae* biosynthesis (44, 47). Possible explanations for the observed activity are that monobromamphenicol is a minor biosynthetic product of *S. venezuelae* and that chloramphenicol was present at greater abundance in a separate fraction. We identified an inhibitory fraction among the HPLC fractions collected. The inhibitory fraction contained chloramphenicol as detected by LC-MS/MS (Fig. S2C and D). We considered the possibility that chloramphenicol is primarily responsible for inducing *B. subtilis* sliding motility but that, upon concentrating the crude extract, the more abundant chloramphenicol achieved an inhibitory concentration while monobromamphenicol reached a stimulatory concentration. To determine whether chloramphenicol induces sliding mobility, the chloramphenicol-containing fraction was serially diluted, and each dilution was tested for activity with *B. subtilis* (Fig. 4A). At a concentration approximately 8-fold lower than that of the parent fraction, chloramphenicol induced a sliding response by *B. subtilis* that was similar to the response seen upon challenge with *S. venezuelae* (Fig. 1B). Concentration-dependent differences in activity are described as hormesis, a phenomenon typically characterized by stimulatory effects of an agent at low doses and inhibitory or toxic effects of the same agent at higher concentrations (32, 33). To determine the corresponding concentration of chloramphenicol that is active for sliding induction, a commercially available source of pure chloramphenicol was serially diluted and added directly into the agar media. We observed the maximal sliding response by *B. subtilis* at 0.3 µg/ml chloramphenicol, which corresponds to an approximate concentration of 1 µM (Fig. 4B; see also Movie S2). These results demonstrate that subinhibitory amounts of chloramphenicol induce a widespread change in a population of *B. subtilis*, leading to mobilization of the colony.

10.1128/mSphere.00586-17.10 MOVIE S2  Induction of *B. subtilis* sliding motility by a subinhibitory concentration of chloramphenicol. (A) A *B. subtilis* population cultured on medium without chloramphenicol for 72 h at 30°C. (B) A *B. subtilis* population cultured on the same medium as that described for panel A with supplementation of 0.3 µg/ml (~1 µM) chloramphenicol. The chloramphenicol induced migration in the form of sliding motility. The agar plates were 8.4 cm in diameter. Download MOVIE S2, MOV file, 1.8 MB. Copyright © 2018 Liu et al. 2018 Liu et al. This content is distributed under the terms of the Creative Commons Attribution 4.0 International license.

### Antibiotics that block translation induce *B. subtilis* sliding motility.

To determine whether the sliding response was specific to chloramphenicol, we selected 14 antibiotics to test for induction primarily on the basis of their different mechanisms of action. Serial dilutions of each antibiotic were spotted on filter discs placed adjacent to *B. subtilis*. In addition to chloramphenicol, three other antibiotics induced sliding mobility. The inducing antibiotics were tetracycline, erythromycin, and spectinomycin, which all target the ribosome and block protein translation (Table 1) (48–51). Interestingly, no aminoglycoside antibiotic tested resulted in activation of sliding mobility by *B. subtilis*, indicating that errors in translation do not trigger the sliding response. These results led us to conclude that *B. subtilis* responds to some types of translation inhibitors at subinhibitory concentrations by activating sliding motility.

**TABLE 1:** Four of 14 tested antibiotics induced sliding^a^

Antibiotic	Mobility inducer	Target
Chloramphenicol	Yes	50S
Spectinomycin	Yes	30S
Erythromycin	Yes	50S
Tetracycline	Yes	30S
Apramycin	No	30S
Kanamycin	No	30S
Gentamicin	No	30S
Lincomycin	No	50S
Hygromycin	No	30S
Phleomycin	No	DNA
Novobiocin	No	DNA gyrase
Ampicillin	No	Transpeptidase
Rifamycin	No	RNA polymerase

a In each case, the tested concentrations ranged from inhibitory levels to levels having no detectable effect.

**FIG 5:**
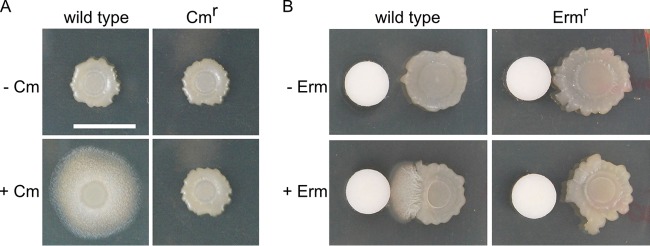
The ribosome plays a key role in antibiotic-induced sliding. (A) Wild-type strain NCIB 3610 and chloramphenicol (Cm)-resistant strain Cm^r^ were spotted on the agar plate in the absence (-) or presence (+) of Cm (0.3 µg/ml). (B) Wild-type strain NCIB 3610 and erythromycin (Erm)-resistant strain Erm^r^ were spotted on the agar plate in the absence or presence of Erm (10 µl of 12.5 µg/ml solution). Pictures were taken at h 24. Filter disc diameter, 0.6 cm. Bar, 1 cm.

To determine whether the sliding response was dependent upon interaction of the antibiotics with the ribosome, as opposed to an unidentified cellular target, we investigated the effect of antibiotic resistance on sliding. First, a chloramphenicol-resistant (Cm^r^) *B. subtilis* strain, which expressed chloramphenicol acetyltransferase, was used to determine whether chemical modification of the antibiotic disrupted sliding. Acetylation of chloramphenicol interferes with binding of the drug to the ribosome (52–54). The Cm^r^ strain did not induce sliding when challenged with chloramphenicol (Fig. 5A). Correspondingly, when wild-type *B. subtilis* was treated with chloramphenicol acetate at a concentration equivalent to the concentration at which chloramphenicol induced sliding, there was no response (Fig. S3A). However, at elevated (4-fold-greater) levels, chloramphenicol acetate induced sliding activity, indicating that the resistance was overcome with greater amounts of the modified antibiotic. Second, to determine whether direct modification of the ribosome prevented sliding mobility, an erythromycin-resistant (Erm^r^) *B. subtilis* strain was treated with inducing concentrations of erythromycin. The Erm^r^ strain expressed a methyltransferase that specifically methylates 23S rRNA, which blocks erythromycin binding (55, 56). In comparison to the wild-type strain results, the Erm^r^ *B. subtilis* strain did not induce sliding in response to erythromycin (Fig. 5B). Collectively, these results suggest that, when present at subinhibitory concentrations, antibiotics that induce sliding motility target the ribosome and presumably cause protein translation stress.

10.1128/mSphere.00586-17.4 FIG S3  Chloramphenicol acetate and lincomycin are inactive for sliding induction. (A) Different amounts of Cm and Cm acetate were spotted on filter discs adjacent to *B. subtilis* colonies. Cm acetate did not induce sliding at an amount (625 ng) equivalent to the amount of Cm that induced sliding. However, Cm acetate induced sliding in a greater amount (2,500 ng), which was equivalent to the amount of Cm that inhibited growth of *B. subtilis*. The solvent control for both Cm and Cm acetate was 10% ethanol (in H_2_O). Pictures were taken at h 24. Filter disc diameter, 0.6 cm. (B) Different amounts of lincomycin (indicated in micrograms per milliliter) were spotted on filter discs adjacent to *B. subtilis* colonies. The negative-control solvent used in the assay was 10% ethanol, and the positive control was 625 ng Cm. Pictures were taken at h 24. Filter disc diameter, 0.6 cm. Download FIG S3, TIF file, 2.5 MB. Copyright © 2018 Liu et al. 2018 Liu et al. This content is distributed under the terms of the Creative Commons Attribution 4.0 International license.

### Induction of *bmrCD* by a subinhibitory concentration of chloramphenicol is consistent with translation stress.

**FIG 6:**
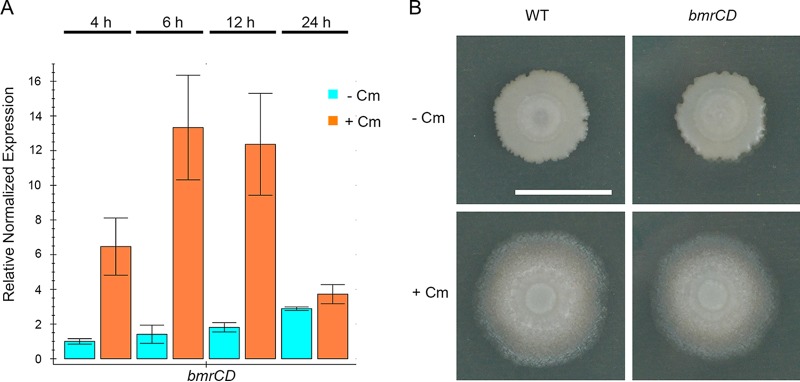
*bmrCD* is related to translation stress but is not required for sliding. (A) Quantitative RT-PCR of *bmrCD* transcript of the wild-type (WT) strain in the absence (-) and presence (+) of chloramphenicol (Cm) at the indicated time points, 4 h, 6 h, 12 h, and 24 h. Quantification cycle (*C*_*q*_) values were normalized to *C*_*q*_ values for *gyrB*. Fold expression values are reported relative to the value for the 4-h sample in the absence of Cm. (B) The WT NCIB 3610 strain and a *bmrCD* deletion strain were spotted on the agar plate in the absence or presence of Cm (0.3 µg/ml). Pictures were taken at h 24. Bar, 1 cm.

Treatment of *B. subtilis* with chloramphenicol and other translation inhibitors at subinhibitory concentrations has been shown to affect gene expression (35, 57, 58). Levels of expression of several genes changed due to chloramphenicol exposure (35). Expression of the *bmrCD* genes, which encode a multidrug efflux transporter, was subsequently shown to be dependent upon the activity of an upstream open-reading frame named *bmrB* (57). The mechanism of expression control couples efficient translation of BmrB to transcription of downstream *bmrCD*, where disruption of translation by inhibitory antibiotics causes enhanced production of BmrCD. However, those prior studies investigated laboratory strains *B. subtilis* 168 and 1A757 in liquid cultures where sliding would not be observed. To determine whether undomesticated *B. subtilis* NCIB 3610 would activate *bmrCD* expression in our sliding assays, the transcript abundance of *bmrCD* was monitored using quantitative reverse transcription-PCR (qRT-PCR). Transcripts of *bmrCD* were elevated a maximum of 12-fold over the untreated control abundance during the initial 12 h of the experiment (Fig. 6A). The peak abundance of *bmrCD* transcript occurred between 6 and 12 h. However, after 24 h, when sliding motility was clearly observed, the *bmrCD* transcript abundance was restored to nearly wild-type levels. This pattern of *bmrCD* expression is consistent with a transient expression pattern observed previously (57). The elevated expression of *bmrCD* indicated that the presence of chloramphenicol at a subinhibitory concentration was stressing protein translation, in accordance with the coupled transcription-translation of *bmrBCD*.

The induced transcription of *bmrCD* is not limited to chloramphenicol. Multiple antibiotics, all targeting the ribosome, were shown to also lead to elevated *bmrCD* expression when used at subinhibitory concentrations (57). Intriguingly, lincomycin was previously shown to induce *bmrCD* expression strongly at subinhibitory concentrations but did not induce sliding at any concentration tested in our assays (Fig. S3B). This observation indicated independence of sliding motility and the effects of translation stress on expression of the BmrCD multidrug efflux pump. To determine whether *bmrCD* induction is required for sliding motility, *bmrC*, *bmrD*, and *bmrCD* mutant strains were challenged with a subinhibitory chloramphenicol concentration. Despite the absence of BmrCD, the sliding response was intact for the mutant strains (Fig. 6B; see also Fig. S4A). Therefore, the *bmrCD* genes are not required for sliding motility. To determine whether disrupting regulation of *bmrCD* would perturb chloramphenicol-induced sliding, we generated a markerless deletion of the *bmrB* open reading frame (ORF), placing the *bmrCD* genes directly under the transcriptional control of the *bmrB* promoter. When exposed to a subinhibitory chloramphenicol concentration, the *bmrB* mutant strain maintained the sliding response, further supporting the conclusion that the *bmrCD* genes are not involved in sliding motility (Fig. S4A). Additionally, the mutant strains were not hypersensitive to chloramphenicol, either for sliding or for growth (Fig. 6B; see also Fig. S4B). These observations suggest that, while elevated *bmrCD* expression indicates translation stress, as-yet-unidentified events are the drivers of antibiotic-induced sliding motility.

10.1128/mSphere.00586-17.5 FIG S4  Phenotypic effects of *bmrBCD* disruptions on sliding and sensitivity. (A) Wild-type (WT) *B. subtilis* NCIB3610 and *bmrB*, *bmrC*, and *bmrD* knockout strains were spotted on the agar plate in the absence or presence of Cm. Pictures were taken at h 24. Bar, 1 cm. (B) Growth curves of the wild-type (WT) and *bmrB*, *bmrC*, *bmrD*, and *bmrCD* knockout strains in response to different concentrations (0, 1, 2, 4, 8, and 16 μM) of Cm in the period of 18 h. Download FIG S4, TIF file, 2.6 MB. Copyright © 2018 Liu et al. 2018 Liu et al. This content is distributed under the terms of the Creative Commons Attribution 4.0 International license.

## DISCUSSION

Through tracking changes in colony morphology and mobility during competition between two species of bacteria, we observed that *S. venezuelae* induces sliding motility in *B. subtilis*. We found that exposure to low doses of monobromamphenicol and chloramphenicol induced mobilization of the *B. subtilis* population. Subsequently, we found that multiple translation-inhibiting antibiotics induced *B. subtilis* sliding. The observed pattern of interaction is indicative of antibiotic hormesis. In this instance, exposure to low doses of translation inhibitory molecules triggers a mobilization of a *B. subtilis* population. The activation of sliding motility may provide a substantial competitive advantage to *B. subtilis*, enabling the cells to relocate rapidly and avoid inhibitory doses of antibiotics. Streptomycetes produce many translation-inhibiting antibiotics, consistent with our observation that sliding is frequently observed using pairings of *Streptomyces* spp. with *B. subtilis* NCIB 3610 (Fig. 1A).

Perception of low doses of toxic or growth-inhibitory substances provides an opportunity for bacteria to activate protective responses. For instance, biofilms provide a specialized niche for inhabitant bacteria, which alter their physiology and expression of resistance functions, and persisters are protected due to their paused growth and metabolism. Two described examples of antibiotic-protective responses are the formation of biofilms and the formation of persister cells, which lend adaptive resistance to the target organism (8, 12, 34, 59, 60). In both cases, the outcomes are cells that become recalcitrant in the presence of antibiotics. Upon exposure to subinhibitory levels of translation stress, the outcome for *B. subtilis* is strikingly different. The cells engage a growth-dependent type of mobility, which provides a means to physically relocate a subpopulation. Thus, instead of preventing growth to avert antibiotic stress, *B. subtilis* activates growth-dependent mobilization. Induced motility in response to antibiotics has rarely been described. Tobramycin was shown previously to enhance the swarming motility of *P. aeruginosa* (22). In contrast, exposure to several antibiotics was found to diminish motility in multidrug-resistant *Salmonella enterica* serovar Typhimurium (5). The observed effects of antibiotics suggest that enhanced motility plays an important role in physiological adaptations of bacteria to antibiotic exposure.

**FIG 7:**
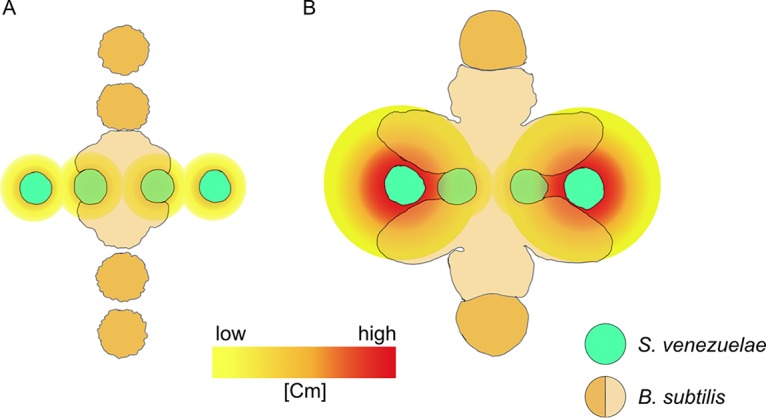
Summary model for concentration-dependent effects of chloramphenicol on *B. subtilis*. The competitive culture format for *S. venezuelae* and *B. subtilis* suggests a model for the spatial and temporal effects of population growth on production and diffusion of chloramphenicol in the agar medium. (A) Early (~24 h) development of the *S. venezuelae* strain (light green spots) results in low concentrations (yellow) of chloramphenicol in the medium, sufficient for stimulating sliding motility in the proximal *B. subtilis* strain (light tan shapes). (B) Continued growth (~48 h) and, presumably, chloramphenicol biosynthesis by the proximal *S. venezuelae* spot are impeded by the migratory population of *B. subtilis*. During this time, the more distal spots of *S. venezuelae* grow to a greater extent and produce higher yields of chloramphenicol. The concentration of chloramphenicol (and possibly other, unidentified metabolites) becomes sufficient (red) to impede growth and progression of the sliding population of *B. subtilis*, which is therefore prevented from contacting the *S. venezuelae* population. The unaffected populations of *B. subtilis* (not mobilized by chloramphenicol exposure) are visible as dark tan spots.

*Bacillus subtilis* displayed counterintuitive directionality with respect to its reaction to chloramphenicol in our assays, which may have additional benefits suggested by the migration pattern relative to *S. venezuelae*. As observed in still and video images of the interaction, the initial response of *B. subtilis* is movement toward the colony of *S. venezuelae*. One speculative idea is that the apparent directionality is a product of the assay format, where cells on the proximal side of a patch are first to respond and expand outward. The outward expansion leads to rapid colonization of the agar surface, including the original spot of *S. venezuelae* (Fig. 7). Although further evidence is required, the interaction pattern suggests that an early expansion of *B. subtilis* populations results in suppression of continued growth of the streptomycete, thereby preventing further production of chloramphenicol. Following several more hours of culture, the *B. subtilis* outward migration extends toward more distantly situated spots of *S. venezuelae*. However, the migratory population is repelled from the *S. venezuelae* spots (Fig. 7). One possible explanation is that additional growth of the streptomycetes results in production of growth-inhibitory amounts of chloramphenicol or other antibiotics. If the patterns do indeed reflect responses to changing antibiotic concentrations, the competitive fitness advantage to early activation of sliding mobility would be dually protective, providing an early opportunity to overtake the competitor and an escape mechanism if antibiotic concentrations reach inhibitory levels.

The mechanism by which subinhibitory antibiotics induce mobilization is likely linked to protein translation. The mechanism of action for each of the inducing antibiotics is that of blocking translation. Intriguingly, the effect is not limited to a single site of action, such as the peptidyl transfer site or the exit tunnel (54, 61). Instead, the mechanisms of activation converge on blockage of progression of translation, as opposed to misincorporation of amino acids or damage to other cellular structures (62–64). This connection is illustrated by the transcriptional activation of *bmrCD* by subinhibitory concentrations of chloramphenicol and other antibiotics. Stalling in translation of BmrB permits the transcription of the *bmrCD* genes (57). Because the *bmrCD* genes are not required for mobilization, sliding induction must require other changes in *B. subtilis* exposed to inducing antibiotics. Further pursuit of changes in transcriptome, proteome, and metabolome analyses will likely uncover key factors that lead from translation stress to sliding mobility for *B. subtilis*.

A growing body of evidence demonstrates the many mechanisms by which bacteria detect antibiotics in the environment and initiate protective responses. These responses include formation of biofilm and persisters, enhanced virulence, motility, and other physiological adaptations. The consequences of bacterial adaptation to low doses of antibiotics are likely to have a substantial impact on bacterial communities. Adaptive changes provide opportunities for bacteria to acquire specific mechanisms of resistance to a given antibiotic or class of antibiotics (23, 65, 66). In addition, adaptive changes that influence specialized metabolism, virulence, and mobility are likely to affect interactions in ways that ripple outward to impact other species in a community and even plant and animal host organisms.

## MATERIALS AND METHODS

### Strains, primers, antibiotics, and growth media.

The strains of *Bacillus subtilis* used in this study are listed in Table S1 in the supplemental material. *Bacillus subtilis* mutant strains in the strain 168 (originally from Bacillus Genetic Stock Center [BGSC]) or strain PY79 background were transduced into NCIB 3610 by SPP1 phage transduction using standard procedures (67). Plasmid pDR244 was used to generate markerless deletions in the *mls*-marked *B. subtilis* NCIB 3610 strains by looping out a *loxP*-flanked macrolide, lincosamide, and streptogramin B (MLS) resistance cassette. To obtain a *bmrCD* double-knockout strain with kanamycin resistance, long-flanking region homology (LFH) PCR was used. Primers *bmrC*-up1000-fwd and *bmrC*-up1000-rev were used to amplify the *bmrC* upstream 1-kb region, and primers *bmrD*-down1000-fwd and *bmrD*-down1000-rev were used to amplify the *bmrD* downstream 1-kb region. Primers *kan*-fwd and *kan*-rev were used to amplify the kanamycin cassette. The primers are listed in Table S2. All antibiotics were purchased from Sigma. *B. subtilis* strains were cultured at 37°C in lysogeny broth (LB) and were inoculated onto GYM7 plates (0.4% [wt/vol] d-glucose, 0.4% [wt/vol] yeast extract, 1.0% [wt/vol] malt extract, 1.5% [wt/vol] agar, 100 mM MOPS [morpholinepropanesulfonic acid], 2.5 mM KH_2_PO_4_, 2.5 mM K_2_HPO_4_, pH 7.0) and grown to an optical density at 600 nm (OD_600_) of 1. *Streptomyces* spore stocks were maintained in water at 4°C. Additional details of the methods used are provided in Text S1 in the supplemental material.

10.1128/mSphere.00586-17.6 TABLE S1  Bacterial strains used in this study. Download TABLE S1, DOCX file, 0.1 MB. Copyright © 2018 Liu et al. 2018 Liu et al. This content is distributed under the terms of the Creative Commons Attribution 4.0 International license.

10.1128/mSphere.00586-17.7 TABLE S2  Primers used in this study. Download TABLE S2, DOCX file, 0.05 MB. Copyright © 2018 Liu et al. 2018 Liu et al. This content is distributed under the terms of the Creative Commons Attribution 4.0 International license.

10.1128/mSphere.00586-17.8 TABLE S3  Transposon mutagenesis results. Download TABLE S3, DOCX file, 0.03 MB. Copyright © 2018 Liu et al. 2018 Liu et al. This content is distributed under the terms of the Creative Commons Attribution 4.0 International license.

### Coculture assays and motility assays.

Coculture assays were performed as previously described (19). Briefly, 2.5 μl of *Streptomyces* spores (10^7^ spores/ml) was spotted in the horizontal line and grown for 12 h at 30°C. A 1.5-μl volume of *B. subtilis* was then spotted 6 mm from a *Streptomyces* sp. in the vertical line. For motility assays, 1.5 μl of *B. subtilis* was spotted 6 mm from wells or filter discs on the agar plate.

### Sliding inducer extraction and identification.

*S. venezuelae* was cultured on the top layer of GYM7 plates separated from the bottom layer by a sheet of cellophane. The top layer (5 ml) along with the cellophane was removed after 5 days of *S. venezuelae* growth. Metabolites were extracted from the lower layer (20 ml) by freezing the agar and separating aqueous media by filtration through a 60-ml syringe containing a layer of Miracloth (EMD Millipore). The squeezed extracts were pooled and then lyophilized. The crude extract was suspended in one-fifth of the original volume in H_2_O. The crude extracts were initially fractionated by the use of an SPE C_18_ column (Sigma). To extract the mobility inducer, 3 ml of crude extract was applied to the 3-ml SPE C_18_ column (Supelco). The column was washed with 6 ml of 10% (vol/vol) methanol, followed by elution with a 20% (vol/vol) stepwise gradient (from 20% to 100%) of methanol/H_2_O. Methanol in all fractions was removed using a rotary evaporator. The concentrated fractions were suspended in 200 µl H_2_O. All fractions were tested for mobility-inducing activity by spotting 10 µl on a well or a filter disc 6 mm away from *B. subtilis* colonies, and the mobility induction was observed after 24 h. The 40% (vol/vol) methanol fraction was active, and multiple 40% (vol/vol) extracts were pooled for further analysis. The 40% (vol/vol) methanol fraction was further fractionated by HPLC (Agilent 1200) using a semipreparative C_18_ column (Phenomenex) (10 by 250 mm, 5-μm particles). An isocratic method was used (30% [vol/vol] solvent A, 70% [vol/vol] solvent B. 20 min in total) with a flow rate of 4 ml/min. Solvent A was acetonitrile. Solvent B was 0.1% (vol/vol) formic acid–H_2_O. For each injection, 100 µl pooled active fraction was applied. Time-based fractions from HPLC were collected and tested for mobility-inducing activity. Those active fractions were analyzed by LC-MS/MS. Specifically, LC-MS/MS was performed with an Agilent 1260 HPLC system coupled with a binary pump and a 1200 series diode array detector UV light-visible light (UV-Vis) detector (compounds were detected at 254 nm, 340 nm, and 420 nm) followed by a MicroTOF-Q Ⅱ mass spectrometer (Bruker Daltonics) using an electrospray ionization (ESI) source. Separation was performed with a Supelcosil LC-18 column (Supelco) (15 cm by 3 mm, 3-µm particles). LC conditions were as follows: *t* = 0 min, 100% A; *t* = 2 min, 100% A; *t* = 12 min, 30% A; *t* = 20 min, 30% A; *t* = 25 min, 100% A; *t* = 35 min, 100% A; *t* = 40 min, 100% A. The flow rate was 400 µl/min. Solvent A was 5 mM ammonium acetate buffer (pH 6.6). Solvent B was 75% (vol/vol) methanol and 25% H_2_O. A mass spectrometer was calibrated with a diluted sodium acetate solution, and six *m*/*z* values (158.9641, 362.9263, 498.9012, 566.8886, 634.8760, and 770.8509) were used for the calibration. The mass spectrometer was operated in positive mode in a mass range from 50 to 1,500 Da. The ion source temperature was maintained at 200°C with 8 eV of ionization energy and 4,500 V of capillary voltage. Helium was used as the collision gas.

### RNA extraction.

Wild-type *B. subtilis* NCIB 3610 was grown to the early stationary phase (OD_600_ = 1) and was inoculated on GYM7 plates with or without 1 μM chloramphenicol, followed by incubation at 30°C. *B. subtilis* colonies at 4 h, 6 h, and 12 h and the outer region of colonies at 24 h were scraped after treatment with 3 ml of stabilization mixture (2-ml RNAprotect Bacteria Reagent [Qiagen] with 1-ml Tris-buffered saline [TBS] buffer) on each plate. The bacterial suspension was transferred to a 15-ml conical tube, subjected to 5 s of vortex mixing, and incubated at room temperature for 5 min. Aliquots (500 µl) were transferred to individual 2-ml Eppendorf tubes. Cell pellets were collected by centrifugation at 17,900 × *g* for 10 min. RNA was isolated as previously described (19). Briefly, cells were lysed with lysis buffer (15 mg/ml lysozyme, 5 mg/ml proteinase K, 100 mM Tris HCl−50 mM EDTA buffer, pH 8.0) and subjected to vigorous vortex mixing for 45 min at ambient temperature. A 1-ml volume of Trizol reagent (Sigma) was added to each sample. RNA was precipitated using standard procedures. RNA samples were cleaned with a Turbo DNA-free kit (Applied Biosystems).

### Quantitative RT-PCR (qRT-PCR).

qRT-PCR was performed as described previously (38). Briefly, 50 ng of total RNA was used as the template for cDNA synthesis with a High-Capacity RNA-to-cDNA kit (Thermo Fisher Scientific). A SsoAdvanced Universal SYBR green Supermix kit (Bio-Rad) and a CFX96 Touch real-time PCR thermocycler (Bio-Rad) were used to perform quantitative PCR as previously described (38). *gyrB* was used as the reference gene. Target abundance was normalized to *gyrB*, and the fold change value was calculated by comparison to the untreated sample at 4 h. Each experiment was repeated three times.
