# Supplementary material for: Antibiotic Stimulation of a Bacillus subtilis Migratory Response
Source: mSphere. 2018 Feb 21;3(1):e00586-17. doi: 10.1128/mSphere.00586-17 (PMC5821984; doi:10.1128/mSphere.00586-17)
Supplement: TEXT S1 [file sph001182478s1.docx]

**METHODS**

To identify genes responsible for chloramphenicol(Cm)-induced sliding motility, a *Bacillus subtilis* strain carrying the pMarA plasmid transposon mutagenesis system(3) (PDS0121), which targets TA dinucleotide. The procedure was performed as previously described(1), but with a different screen assay. A single transposition event in each individual cell occurs during growth because pMarA is a single copy plasmid containing a *Himar1* transposase gene controlled by housekeeping sigma factor σ^A^-dependent promoter. PDS0121 strain was grown at 22 ℃ overnight in the presence of kanamycin (5 μg/mL). The overnight culture was diluted to OD_600_=0.05, followed by growth to OD_600_=0.3-0.4 at 22 ℃. Then the culture was grown to OD600=1 (~10^8^ cells/mL) by shifting from 22 ℃ to 42 ℃ to prevent replication of pMarA. The culture at OD_600_=1 represents a library of transposon-insertion mutants.

The culture was diluted 10^6^ fold and plated on agar GYM7 plates with 1 μM Cm. Those colonies that did not exhibit sliding motility were selected, followed by a second round of screen on the same type of medium.

**REFERENCES**

1. Stubbendieck RM, Straight PD. 2015. Escape from Lethal Bacterial Competition through Coupled Activation of Antibiotic Resistance and a Mobilized Subpopulation. PLoS Genet 11:e1005722.

2. Kearns DB, Losick R. 2004. Swarming motility in undomesticated *Bacillus subtilis*. Mol Microbiol 49:581–590.

3. Le Breton Y, Mohapatra NP, Haldenwang WG. 2006. In Vivo Random Mutagenesis of *Bacillus subtilis* by Use of TnYLB-1, a mariner-Based Transposon. Appl Environ Microbiol 72:327–333.
