# Supplementary figures and images for: Antibiotic Stimulation of a Bacillus subtilis Migratory Response
Source: mSphere. 2018 Feb 21;3(1):e00586-17. doi: 10.1128/mSphere.00586-17 (PMC5821984; doi:10.1128/mSphere.00586-17)

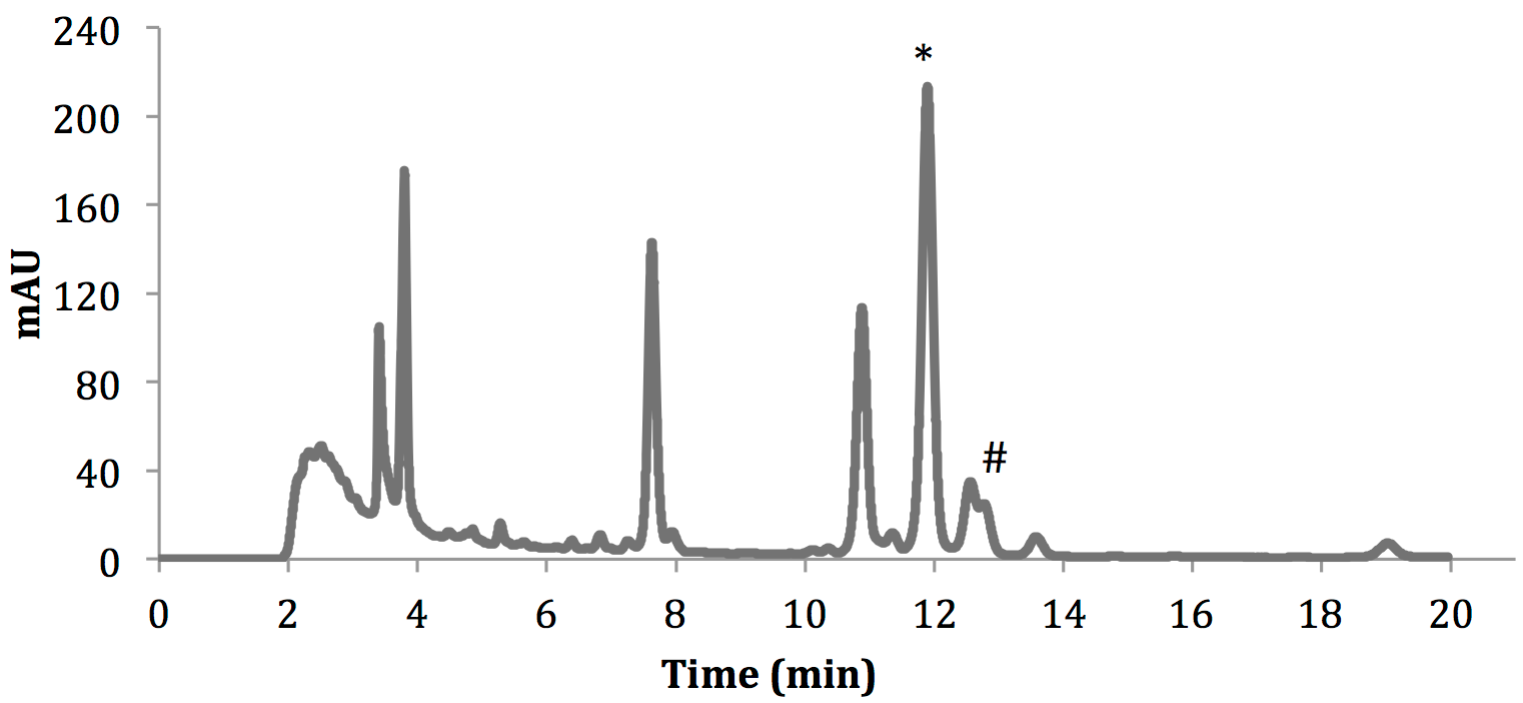

Supplement: FIG S1 [file sph001182478sf1.tif]

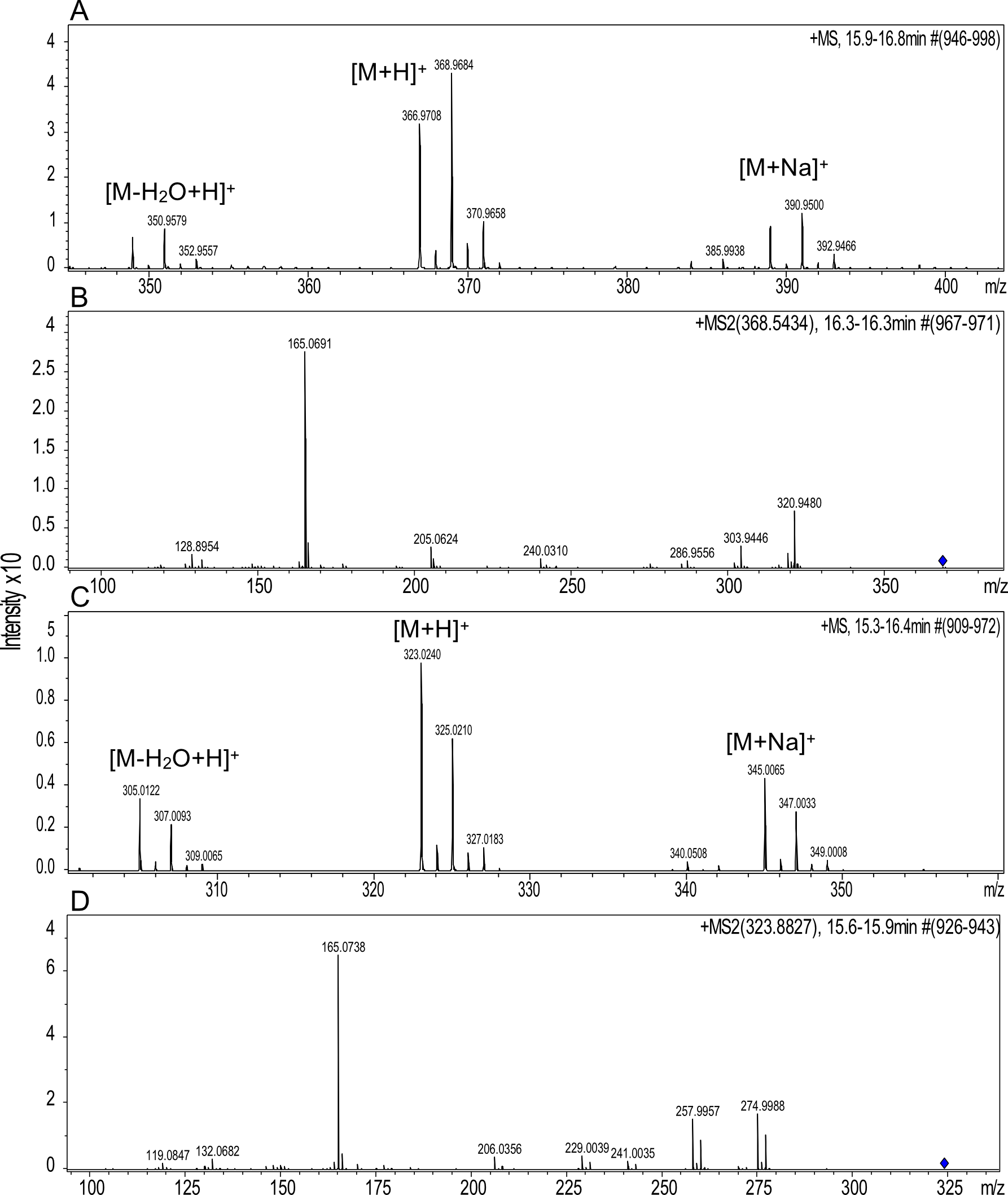

Supplement: FIG S2 [file sph001182478sf2.tif]

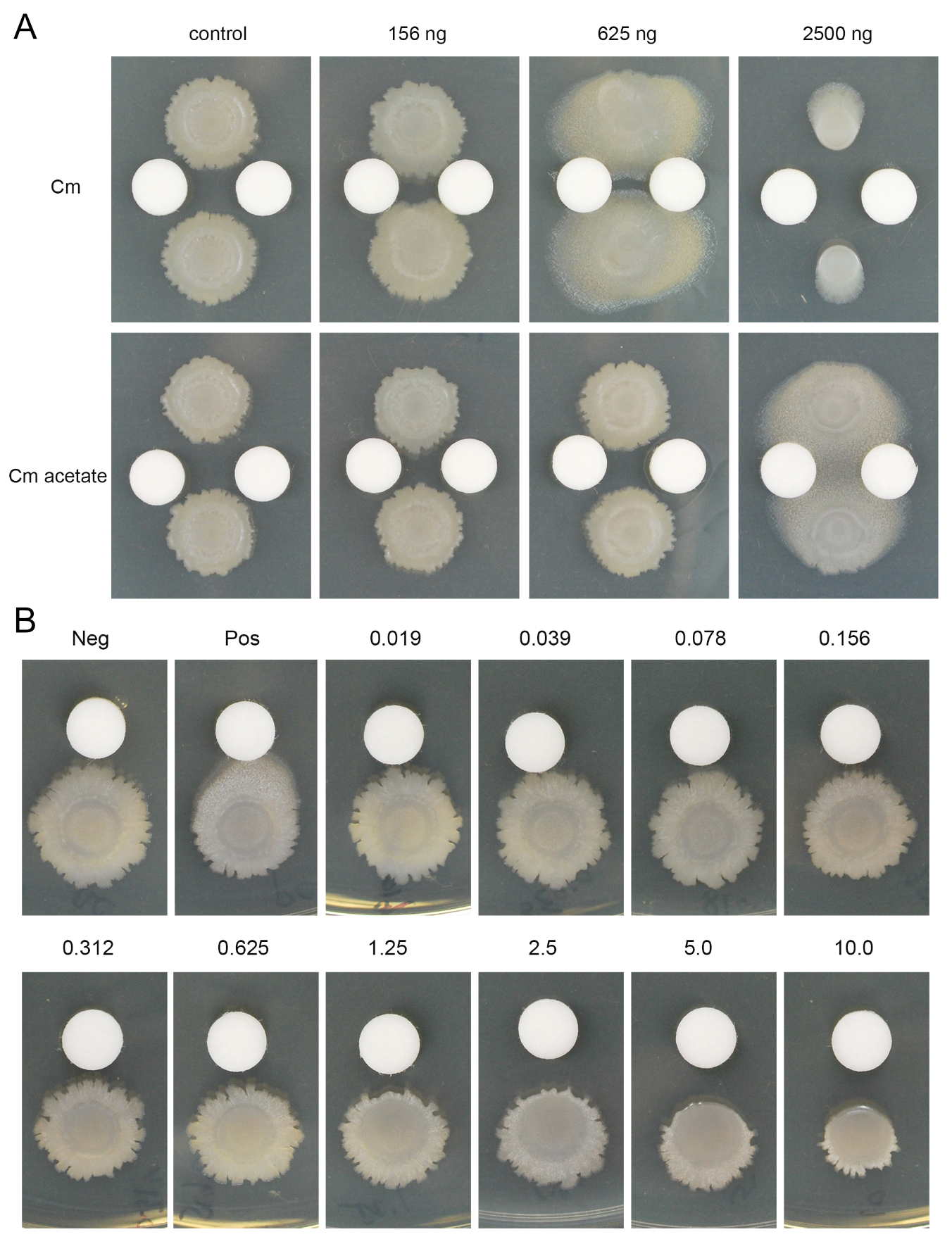

Supplement: FIG S3 [file sph001182478sf3.tif]

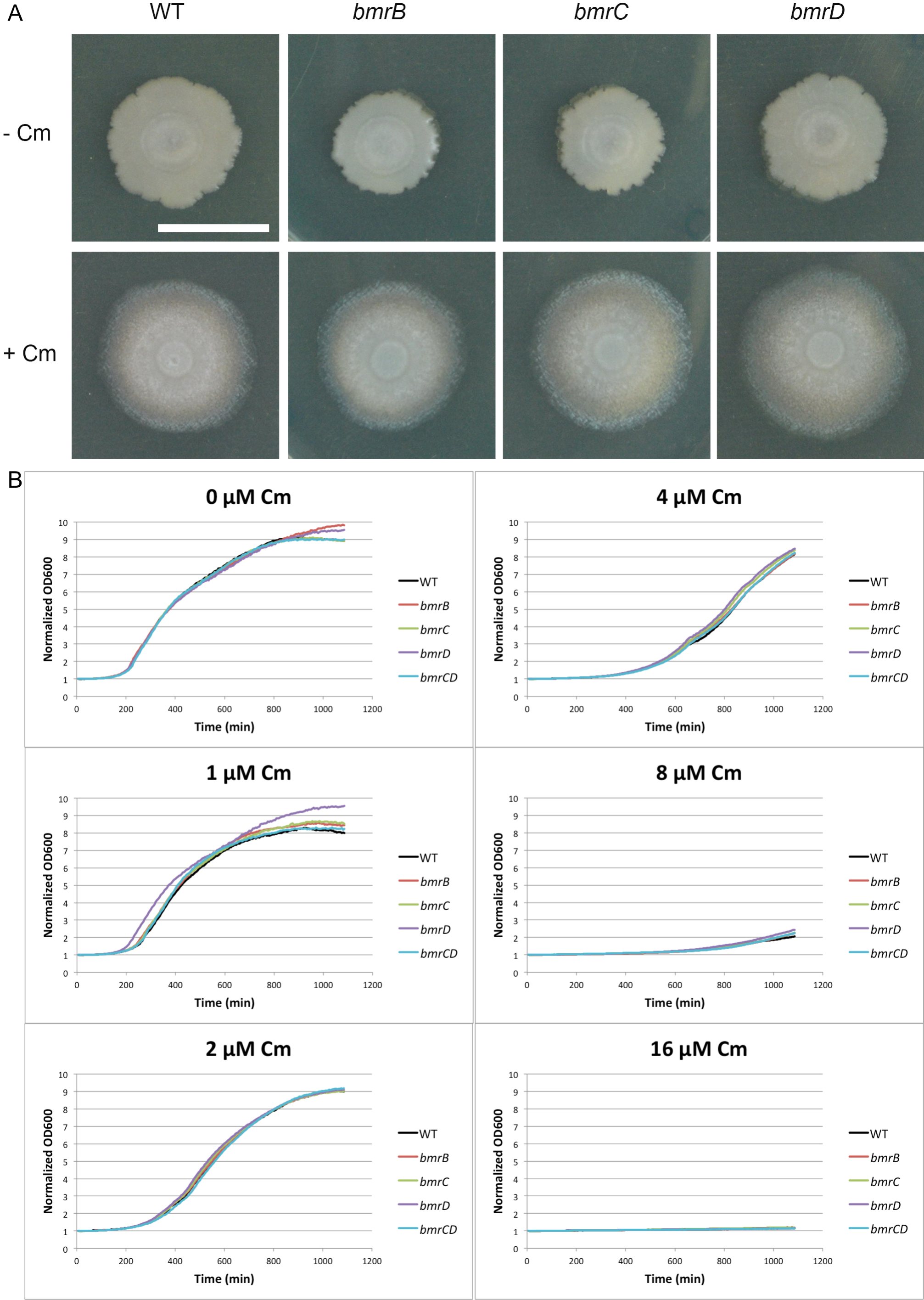

Supplement: FIG S4 [file sph001182478sf4.tif]
