# Supplementary material for: Antibiotic Stimulation of a Bacillus subtilis Migratory Response
Source: mSphere. 2018 Feb 21;3(1):e00586-17. doi: 10.1128/mSphere.00586-17 (PMC5821984; doi:10.1128/mSphere.00586-17)
Supplement: TABLE S1 [file sph001182478st1.docx]

| **Strain** | **Genotype** | **Source** |
| --- | --- | --- |
| PDS0066 | *B. subtilis* NCIB 3610 wild type | Laboratory collection |
| PDS0611 | *B. subtilis* NCIB 3610 Δ*epsH*::*kan* | R.M. Stubbendieck (1) |
| PDS0060 | *B. subtilis* NCIB 3610 Δ*srfAA*::*mls* | (2) |
| PDS0932 | *B. subtilis* NCIB 3610 Δ*hag* | This study |
| PDS0934 | *B. subtilis* NCIB 3610 Δ*bmrCD*::*kan* | This study |
| PDS0939 | *B. subtilis* NCIB 3610 Δ*bmrB* | This study |
| PDS0940 | *B. subtilis* NCIB 3610 Δ*bmrC* | This study |
| PDS0941 | *B. subtilis* NCIB 3610 Δ*bmrD* | This study |
| PDS0207 | *B. subtilis* NCIB 3610 Cm^R^ (*amyE*::P*_pksG_*-*yfp* (*cat*)) | Laboratory collection |
| PDS0234 | *B. subtilis* NCIB 3610 Erm^R^ (Δ*yxe*::*mls*) | Laboratory collection |
| PDS0252 | *Streptomyces venezuelae* ATCC 10712 | John Innes Centre |
| PSK0531 | *Streptomyces lividans* TK24 | Laboratory collection |
| PSK0028 | *Streptomyces coelicolor* M145 | Laboratory collection |
| PDS0116 | *Streptomyces aizunensis* NRRL B-11277 | Laboratory collection |

1. Stubbendieck RM, Straight PD. 2015. Escape from Lethal Bacterial Competition through Coupled Activation of Antibiotic Resistance and a Mobilized Subpopulation. PLoS Genet 11:e1005722.

2. Kearns DB, Losick R. 2004. Swarming motility in undomesticated *Bacillus subtilis*. Mol Microbiol 49:581–590.
