# Supplementary material for: Antibiotic Stimulation of a Bacillus subtilis Migratory Response
Source: mSphere. 2018 Feb 21;3(1):e00586-17. doi: 10.1128/mSphere.00586-17 (PMC5821984; doi:10.1128/mSphere.00586-17)
Supplement: TABLE S2 [file sph001182478st2.docx]

| **Primer** | **Sequence(5’-3’)** |
| --- | --- |
| *gyrB*-qPCR-fwd | GGGCAACTCAGAAGCACGGACG |
| *gyrB*-qPCR-rev | GCCATTCTTGCTCTTGCCGCC |
| *bmrC*-qPCR-fwd | ATTATCCCGCTTCCAGTCAT |
| *bmrC*-qPCR-rev | TTCCAGCACTCTGTCATTCA |
| *bmrC*-up1000-fwd  *bmrC*-up1000-rev  *bmrD-*down1000-fwd  *bmrD*-down1000-rev  *kan*-fwd  *kan*-rev | GACAACACTTAAAAACAGCGGG  GCCAAGCTTTTTCAAAACTGAAAACAT  CGCTCAAAAACCCAAAACAATCG  CGGGGTGTCATTCATCACC  CAGCGAACCATTTGAGGTGATAGG  CGATACAAATTCCTCGTAGGCGCTCGG |
