# Supplementary material for: Antibiotic Stimulation of a Bacillus subtilis Migratory Response
Source: mSphere. 2018 Feb 21;3(1):e00586-17. doi: 10.1128/mSphere.00586-17 (PMC5821984; doi:10.1128/mSphere.00586-17)
Supplement: TABLE S3 [file sph001182478st3.docx]

| Function disrupted | Number | Frequency |
| --- | --- | --- |
| Surfactin or EPS mutants | 6 | 0.0575% |
| *sigH* | 1 | 0.0096% |
| Unrecovered | 2 | 0.0192% |
| Total number | 10430 | |
